# Exploring Potential Regulatory Anesthetic Drugs Based on RNA Binding Protein and Constructing CESC Prognosis Model: A Study Based on TCGA Database

**DOI:** 10.3389/fsurg.2022.823566

**Published:** 2022-04-05

**Authors:** Ying Zheng, Xiao Wen Meng, Jian Ping Yang

**Affiliations:** Department of Anesthesiology, First Affiliated Hospital of Soochow University, Suzhou, China

**Keywords:** cervical squamous cell carcinoma (CESC), bioinformatics, narcotic drugs, predictors, risk score

## Abstract

**Objective:**

To investigate the differential expression of RBPs in cervical squamous cell carcinoma (CESC), analyze the regulatory effect of narcotic drugs on RBPs, and establish the prognostic risk model of CESC patients.

**Methods:**

RNA-SEQ data and clinical case data of cancer and normal samples from CESC patients were obtained from the Cancer Genome Atlas (TCGA) database and Genotype-Tissue Expression (GTEx) database. Differentially expressed RBPs were screened by R language and enriched. The CMAP database is used to predict the anesthetic drugs that regulate the differential expression of RBPs. The prognostic risk score model was constructed by COX regression analysis. Risk score of each CESC patient was calculated and divided into high-risk group and low-risk group according to the median risk score. The prediction efficiency of prognostic risk model was evaluated by Kaplan-Meier (KM) analysis and receiver operating characteristic (ROC) curve, and the correlation between prognostic risk model and clinical characteristics was analyzed. Immunohistochemistry was used to detect the expression of RNASEH2A and HENMT1 in tissues.

**Results:**

There were 65 differentially expressed RBPs in CESC. Five anesthetics, including benzocaine, procaine, pentoxyverine, and tetracaine were obtained to regulate RBPs. Survival analysis showed that seven genes were related to the prognosis of patients, and the CESC risk score model was constructed by COX regression. The risk score can be used as an independent prognostic factor. RNASEH2A and HENMT1 are up-regulated in tumors, which can effectively distinguish normal tissues from tumor tissues.

**Conclusion:**

It is found that different anesthetic drugs have different regulatory effects on the differential expression of RBPs. Based on the differentially expressed RBPs, the prognostic risk score model of CESC patients was constructed. To provide ideas for the formulation of individualized precise anesthesia scheme and cancer pain analgesia scheme, which is helpful to improve the perioperative survival rate of cancer patients.

## Introduction

Cervical cancer is the fourth most common cancer among women in the world, with a high mortality rate among women in developing countries (1). As the most common tissue type of cervical cancer, cervical squamous cell carcinoma (CESC) is a serious threat to women's health, causing about 273,200 death every year (2). In recent years, with the development of cancer screening and various treatment methods such as surgery, radiotherapy and chemotherapy, the clinical prognosis of CESC has been improved to some extent. However, due to the lack of effective diagnostic methods in the early stage of the disease, the risk of metastasis and recurrence of CESC is still high and the prognosis is poor. More and more evidence shows that the abnormal expression of a variety of genes is involved in the occurrence and development of CESC (3–5). In view of the high incidence rate and high mortality rate of CESC, early detection and risk assessment are particularly important for improving the prognosis of CESC patients. Therefore, it is necessary and urgent to find new biomarkers for diagnosis, prognosis and treatment to improve the survival rate of cervical cancer patients. RNA binding proteins (RBPs) are proteins that interact with many types of RNA, including rRNAs, ncRNAs, snrnas, miRNAs, mRNAs, tRNAs and snoRNAs. So far, more than 1,500 RBPs genes have been found in the human genome (6). These RBPs play an important role in maintaining the physiological balance of cells, especially in the process of development and stress response. RBPs can bind to target RNA in a structure or sequence dependent manner to form RNA protein complexes, and regulate mRNA stability, RNA processing, splicing, localization, output and translation at the post transcriptional level (7). In the past decades, many studies have revealed that RBPs are abnormally expressed in tumors, affect the transformation of mRNA to protein, and participate in tumorigenesis (8–10). Among them, only a few RBPs have been deeply studied and found to play a key role in human cancer (11–13). The systematic functional study of RBPs will help us to fully understand its role in tumors.

Narcotic drugs are prescription drugs for the treatment of cancer pain. If they are used continuously, they will cause extreme physical and mental dependence, and can only be used in medical treatment and scientific research. Local anesthetics are commonly used for postoperative analgesia and local anesthesia at the surgical site of cancer patients. Local anesthesia includes intestinal nerve block, local infiltration anesthesia, surface anesthesia and so on. Studies have found that local anesthesia can reduce the stress response after surgery and reduce the inhibitory effect of stress response on the immune system (14). Local anesthesia can reduce the dosage of opinions, reduce the inhibition of opiate analgesics on the immune system, and play a certain role in tumor recurrence and metastasis. At the same time, intestinal nerve block combined with propofol can reduce interleukin (IL) 1 β/ IL-8, increase IL-10. IL-1 β/ IL-8 is considered to be a cytokine promoting tumor formation, and IL-10 is a cytokine inhibiting tumor formation (15). Local anesthetics are commonly used in the clinic. Local anesthesia is also a commonly used anesthesia technology in clinic. Some narcotic drugs inhibit tumor growth, invasion and metastasis. Other narcotic drugs promote tumor growth, invasion and metastasis. Their mechanism may be related to regulating the immune ability of the body to the tumor. So choosing different anesthetic drugs in different preoperative periods may have different effects on tumor recurrence and invasion, and directly affect the prognosis of surgical patients. Therefore, the effects of narcotic drugs on tumors and their related mechanisms need to be further studied and discussed.

Based on the above, the RNA sequencing and clinicopathological data of CESC were downloaded from the Cancer Genome Atlas (TCGA) database and Genotype-Tissue Expression (GTEX) database. Subsequently, abnormally expressed RBPs between CESC and normal cervical tissues were identified by high-throughput bioinformatics analysis, and their potential functions and molecular mechanisms were systematically explored. This study identified some RBPs that may affect the prognosis of CESC and promoted the understanding of the molecular mechanism of CESC progression. These RBPs may provide potential biomarkers for diagnosis and prognosis.

## Materials and Methods

### Data Download and Processing

Three hundred and nine human cervical cancer gene expression samples were downloaded from TCGA database (https://portal.gdc.cancer.gov/), including 3 normal samples and 306 tumor samples, and the corresponding clinical information was provided. The data of the additional 19 normal tissue samples were from GTEx database (https://gtexportal.org/home/datasets). RBPs were collected by Merkley et al. (16) and a total of 1542 RBPs genes were obtained (Supplementary Table 1). The original data were preprocessed with limma software package, and the differentially expressed RBPs were included with error detection rate (FDR) < 0.01 and |logFC (foldchange)| > 2. Differentially expressed RBPs were submitted to the STRING database to identify protein-protein interaction information.

### GO Enrichment and KEGG Pathway Analysis

The biological functions of these differentially expressed RBPs were comprehensively detected by Gene Ontology (GO) enrichment and Kyoto Encyclopedia of Genes and Genomes (KEGG) pathway analysis. GO analysis terms include cellular components (CC), molecular functions (MF), and biological processes (BP). R packages such as clusterProfiler and pathview are used for GO and KEGG pathway enrichment analysis. The difference was statistically significant (*p*.adjust < 0.05).

### Exploration of Anesthetic Drugs With Potential Regulatory Effect

Connectivity map (CMAP) database was used to find anesthetic drugs that regulate the differential expression of RBPs. The database can use computer simulation methods to predict potential drugs that may induce or reverse biological states encoded by gene expression characteristics. Differentially expressed RBPs were classified into up-regulated group and down-regulated group and uploaded to CMAP database. The negative correlation score indicates that the drug inhibits the expression of up-regulated genes and promotes the expression of down-regulated genes, which may reverse the cancer process.

### Screening of Prognostic Genes of RBPs and Construction of Prognostic Model

According to the amount of single gene expression, the median gene expression was used as the grouping method. Prognostic differences of different groups were analyzed by Kaplan-Meier survival analysis. *P*-value and hazard ratio (HR) with 95% confidence interval (CI) were obtained by log-rank test and univariate COX proportional hazards regression. Then, based on the differentially expressed RBPs related to prognosis, the risk score model was constructed by multivariate COX regression analysis, and the risk score was calculated. Calculation formula of prognosis model: riskscore = b1 × Exp1+ b2 × Exp2+ bi × Expi. Among them b represents the coefficient value, and Exp represents the gene expression level. In order to verify the prognostic value of RBPs, the risk score of each CESC sample in TCGA-CESC data was calculated based on the formula. In each data set, the samples were divided into high-risk and low-risk groups by setting the median of risk score as the critical standard. Log-rank test was used to compare the difference in overall survival (OS) between the two groups. In addition, ROC curve analysis was performed using “survivalROC” package to evaluate the prediction ability of the above model. Finally, the nomogram was drawn using RMS package to predict the survival time of patients.

### Correlation Analysis of Independent Prognosis and Clinical Characteristics of Model

Taking the mean value (46.89) as the boundary value, the age was divided into two groups <47 years old and ≥47 years old. Age, gender, grade, stage, T stage and N stage were used as clinical classification variables. Chi square test was used to compare the differences between high and low risk groups. The difference was statistically significant (*P* < 0.05). Univariate and multivariate COX regression models were used to evaluate the relationship between clinical variables, risk score and prognosis, so as to judge whether the risk model can be used as an independent prognostic factor.

### Expression of Model Genes in the Database

In order to determine the expression of model genes in cervical cancer, we used the expression data of cervical cancer and normal tissues in TCGA and GTEx databases to verify the gene expression. Student's *t*-test and Welch's *t*-test were used to analyze the difference of gene expression between cancer and normal tissues. Pearson test was used to analyze the correlation between genes. *P* < 0.001 was considered as significant correlation.

### Immunohistochemistry Staining

With the approval of the hospital ethics committee, paraffin sections of surgically removed tissues of 150 patients with cervical cancer treated from 2017 to 2019 were collected from the pathology department of our hospital, including cervical cancer tissues and corresponding adjacent tissue sections. After paraffin embedding, it was continuously sliced and fixed with formaldehyde. After hydration, it was allowed to stand at 37.5°C for 0.5 h, and 1% Ethylene Diamine Tetraacetic Acid (EDTA) solution was added to block the goat serum. First antibody (1: 300 dilution) and second antibody were added successively, incubated at room temperature, and washed with PBS for 3 times. Add 50 drops per slice μl DAB developer freshly prepared and washed with running water. Counterstain with hematoxylin, dehydrate with alcohol, and seal the film with neutral balsam after drying. Light yellow to brownish yellow is positive. According to the staining intensity, it is divided into (0–1 points) negative, (1–2 points) weak positive, (2–3 points) moderate and (3–4 points) strong positive.

### Correlation Analysis Between RNASEH2A, HENMT1 and Clinical Markers of Cervical Cancer

The correlation between RNASEH2A and HENMT1 and cervical cancer tumor marker (MKI67) was analyzed by GEPIA database. Immunohistochemical staining of RNASEH2A and HENMT1 was performed on microarrays constructed from cancer and adjacent tissues of 150 patients with cervical cancer. In the RNASEH2A protein expression microarray, 120 pairs of RNASEH2A expression tissue nodes of cancer and adjacent tissues were complete. In the HENMT1 protein expression microarray, 93 pairs of HENMT1 expression tissue nodes of cancer and adjacent tissues were complete. Among 150 patients with cervical cancer, the results of MKI67 immunohistochemistry were collected from 115 patients. *T*-test was used to analyze the difference of gene expression between cancer and adjacent cancer. Correlation test is used to reflect the linear correlation between the expression of two genes. ROC curve was used to analyze the diagnostic effect of gene expression on cervical cancer.

## Results

### RBPs in Differentially Expressed CESC Tissues Were Screened

**Figure 1:**
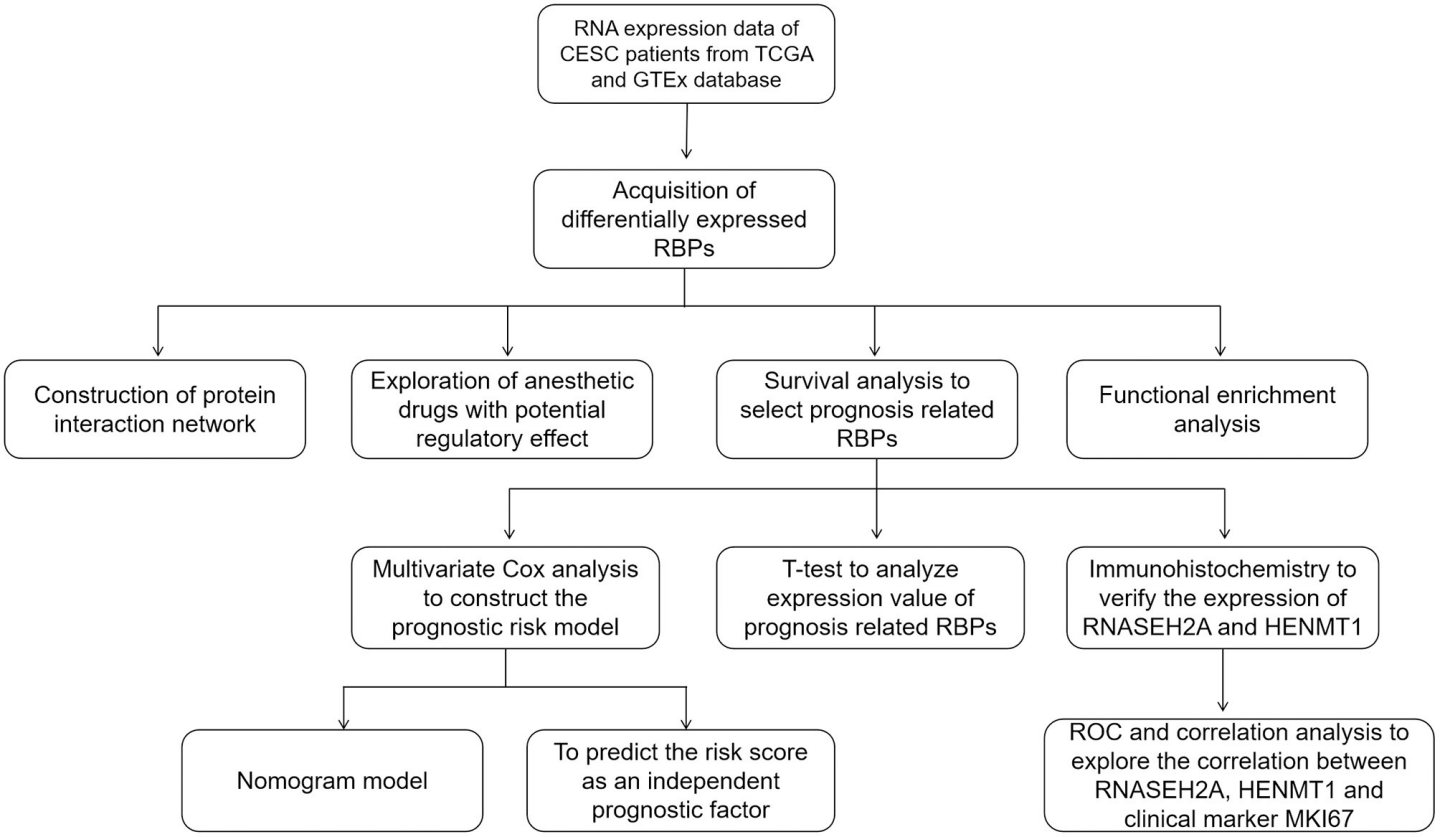
The whole process of exploring potential regulatory anesthetic drugs based on RNA binding protein and constructing CESC prognosis model.

**Figure 2:**
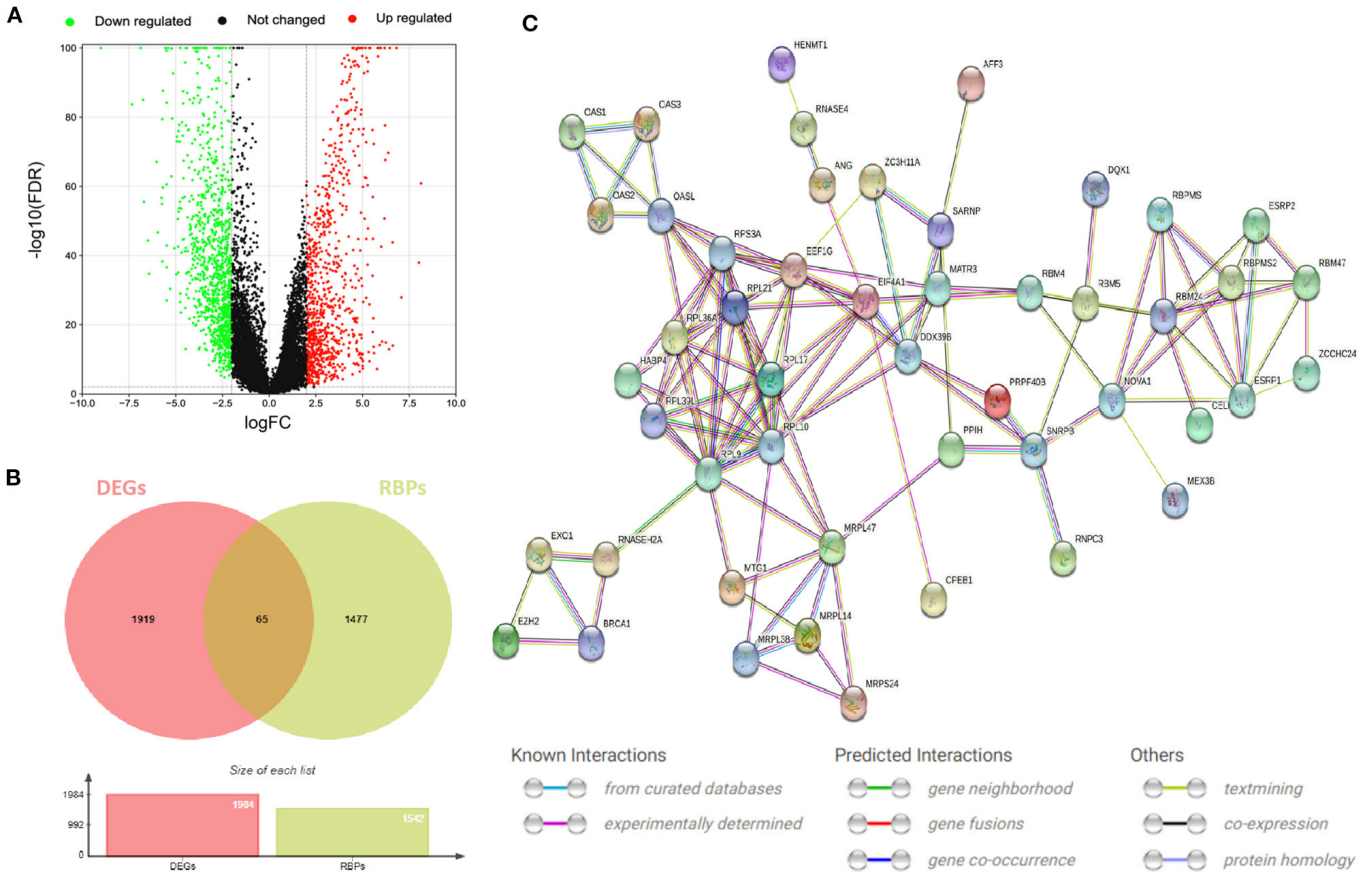
RBPs differentially expressed in CESC. **(A)** Volcano map of DEGs differentially expressed between CESC and normal tissues. The selection criteria were |logFC| > 2 and FDR value <0.01 (red and green represent up-regulated and down-regulated genes, respectively). **(B)** Wayne diagram of differentially expressed RBPs in CESC. **(C)** Protein interaction network differentially expressing RBPs.

The flow of this study is shown in Figure 1. The data obtained from TCGA and GTEx databases are processed by Perl and R language, and 1984 DEGs (Figure 2A) are obtained by “limma” package analysis, including 65 RBPs (Figure 2B). RBPs were collected by Merkley et al. (16) and a total of 1542 RBPs genes were obtained (Supplementary Table 1). STRING database analysis showed that there was a relationship of protein interaction among 49 RBPs (Figure 2C).

### Expression of Differentially Expressed RBPs and Enrichment Analysis of Go and KEGG Pathways

**Figure 3:**
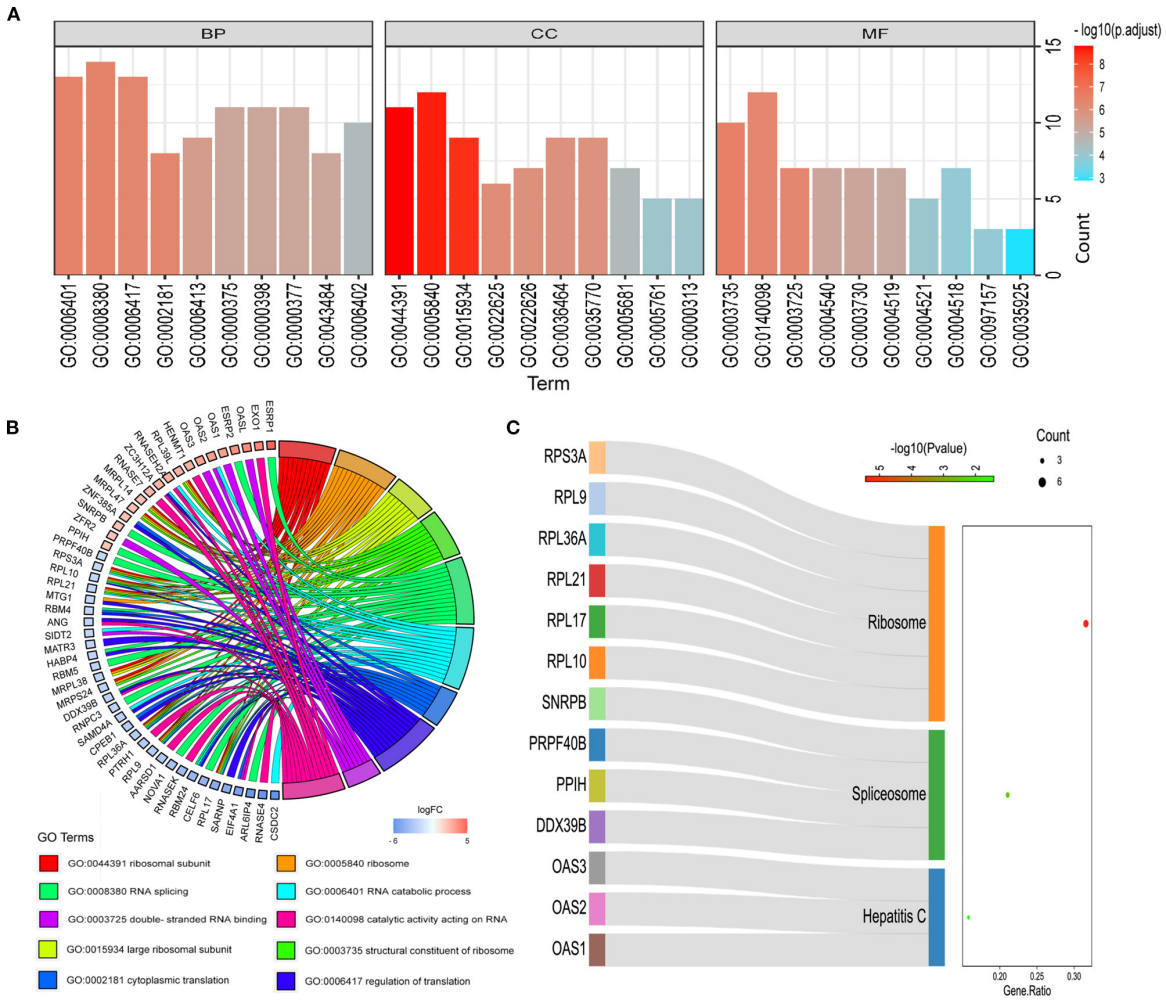
Functional enrichment analysis of differentially expressed RBPs. **(A)** GO analysis divided RBPs into three functional groups: biological process (BP), cell composition (CC) and molecular function (MF). **(B)** Distribution of RBPs in the first 10 GO enrichment functions. **(C)** KEGG analysis of differentially expressed RBPs.

In order to study the function and mechanism of the identified RBPs, these differentially expressed RBPs were enriched and analyzed. The GO analysis results were included in the analysis with *P* < 0.05 as the standard, and the results were divided into BP, CC and MF groups (Figure 3A). The first 10 significant GO analyses showed that RBPs were mainly involved in ribosomal subunit, ribosome, large ribosomal subunit, structural constituent of ribosome, RNA splicing, RNA catabolic process, cytoplasmic translation, regulation of translation, double-stranded RNA binding and catalytic activity acting on RNA (Figure 3B). KEGG signaling pathway is mainly enriched in Hepatitis C, Spliceosome and Ribosome (Figure 3C).

### Potential Small Molecule Drug Screening

**Figure 4:**
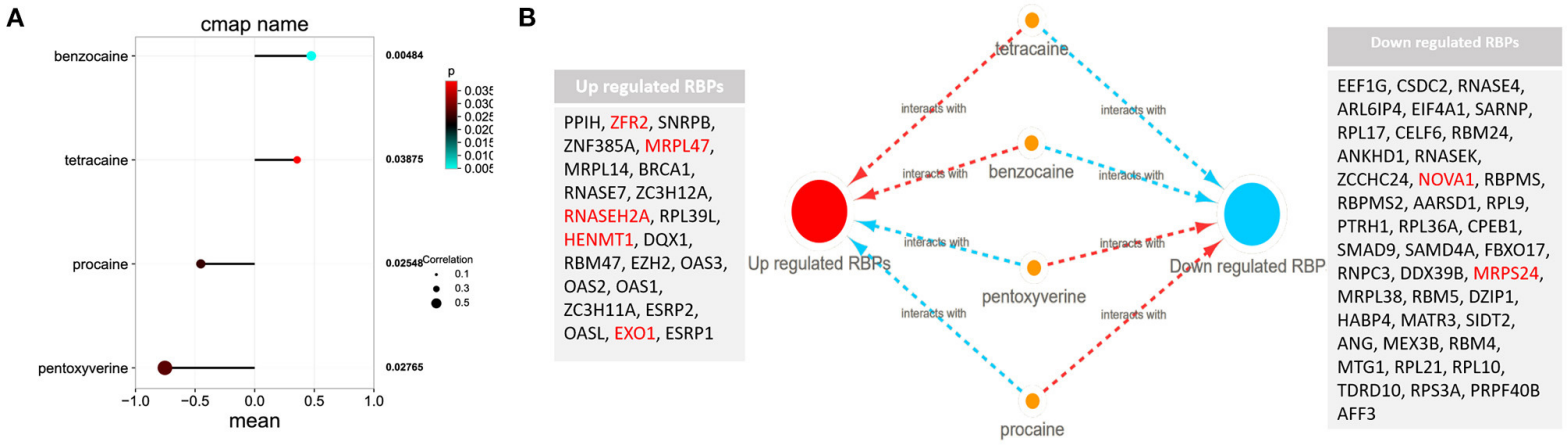
Potential anesthetic drugs that regulate differentially expressed RBPs. **(A)** Regulatory relationship between narcotic drugs and differentially expressed RBPs. Mean indicates the regulatory relationship coefficient (>0 indicates a positive correlation with gene expression) **(B)** the relationship between narcotic drugs and up and down regulated RBPs. The red line represents promoting gene expression and the blue line represents inhibiting gene expression. EXO1, HENMT1, RNASEH2A, MRPL47, ZFR2, MRPS24 and NOVA1 are related to the prognosis of CESC.

Among these highly significantly related molecules, benzocaine, procaine, Pentoxyverine and tetracaine are narcotic drugs (Figure 4A). Procaine and Pentoxyverine are negatively correlated with RBPs gene expression and have potential therapeutic effects on CESC (Figure 4B).

### Construct a Prognostic RBP Prediction Model

**Figure 5:**
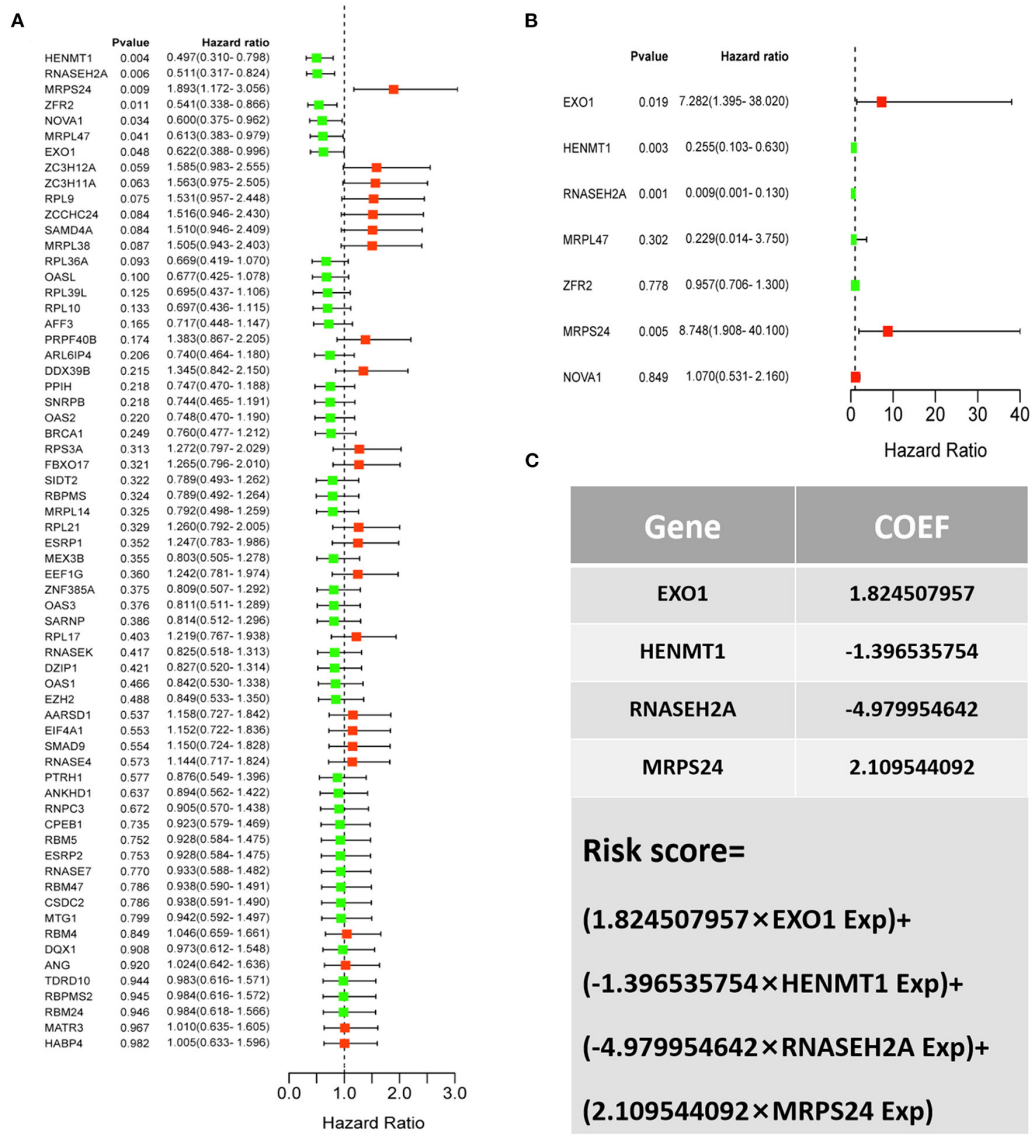
CESC patient risk model based on prognosis related RBPs **(A)** Survival analysis was used to screen prognosis related RBP. **(B)** Multivariate prognostic analysis of seven prognosis related RBPs (EXO1, HENMT1, RNASEH2A, MRPL47, ZFR2, MRPS24 and NOVA1). **(C)** Calculation formula of comprehensive risk score of four genes. Exp stands for gene expression.

For the survival analysis of 65 differentially expressed RBPs, the *P*-value and hazard ratio (HR) with 95% confidence interval (CI) were obtained by logrank test and univariate COX proportional hazards regression (Figure 5A). According to the results of survival analysis, seven RBPs (EXO1, HENMT1, RNASEH2A, MRPL47, ZFR2, MRPS24 and NOVA1) were related to the prognosis of patients. Subsequently, seven prognostic RBPs were analyzed by multiple COX regression, in which EXO1, HENMT1, RNASEH2A and MRPS24 can be used as independent predictors of CESC prognosis (Figure 5B). Then, the prognostic risk model of CESC was constructed with the above four genes. The risk score of each sample was calculated according to the risk coefficient and the expression of 4 RBPs (Figure 5C).

### Risk Model Performance Evaluation

**Figure 6:**
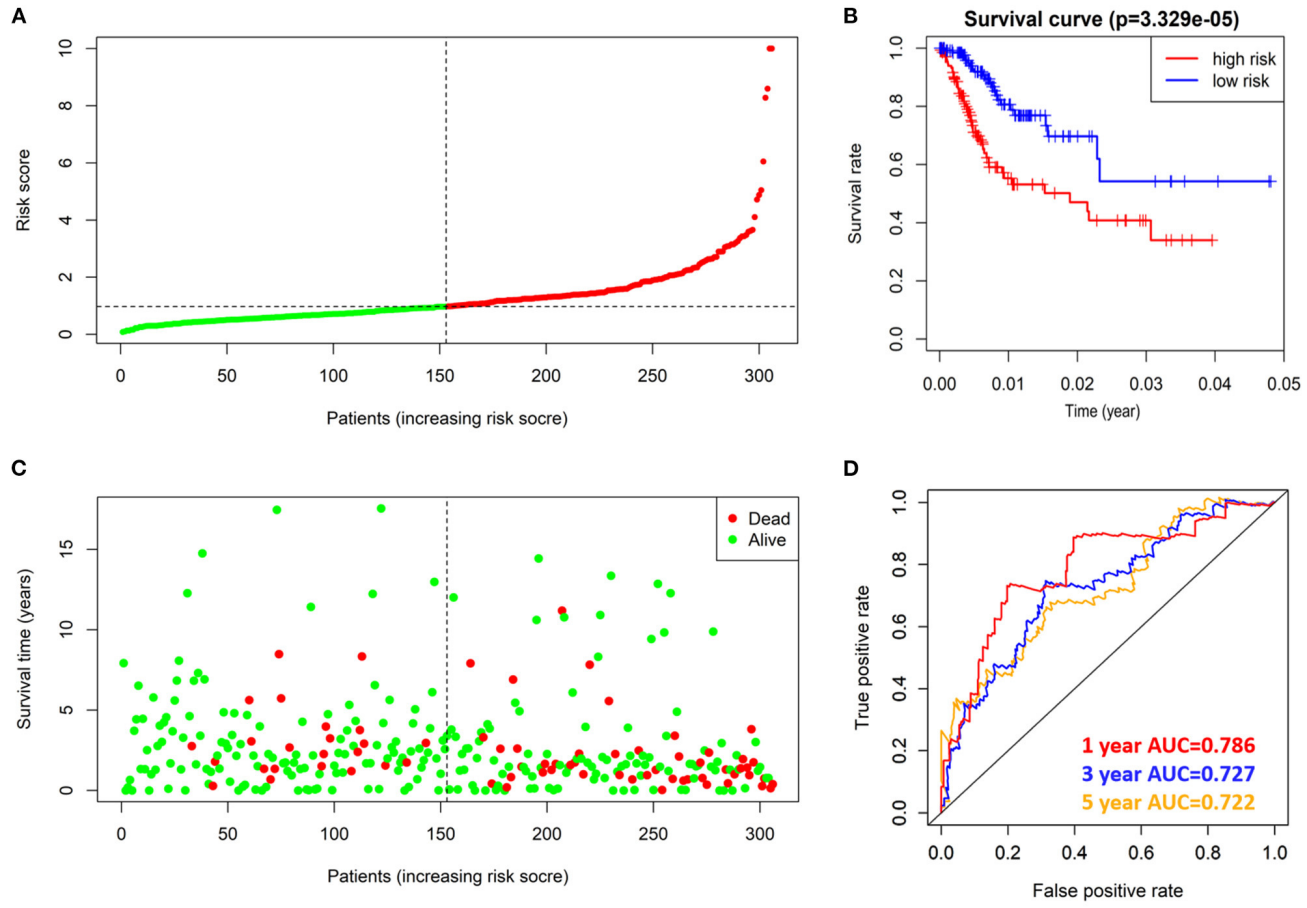
prognostic value of 4 prognostic related RBPs risk models in TCGA dataset. **(A)** Distribution of risk scores in prognosis related RBPs models. **(B)** Kaplan-Meier survival curve of OS in high-risk group and low-risk group. **(C)** Different patterns of survival status and survival time between high-risk group and low-risk group. **(D)** ROC to evaluate the prognostic efficacy of risk score in predicting patients at 1, 3 and 5 years.

**Figure 7:**
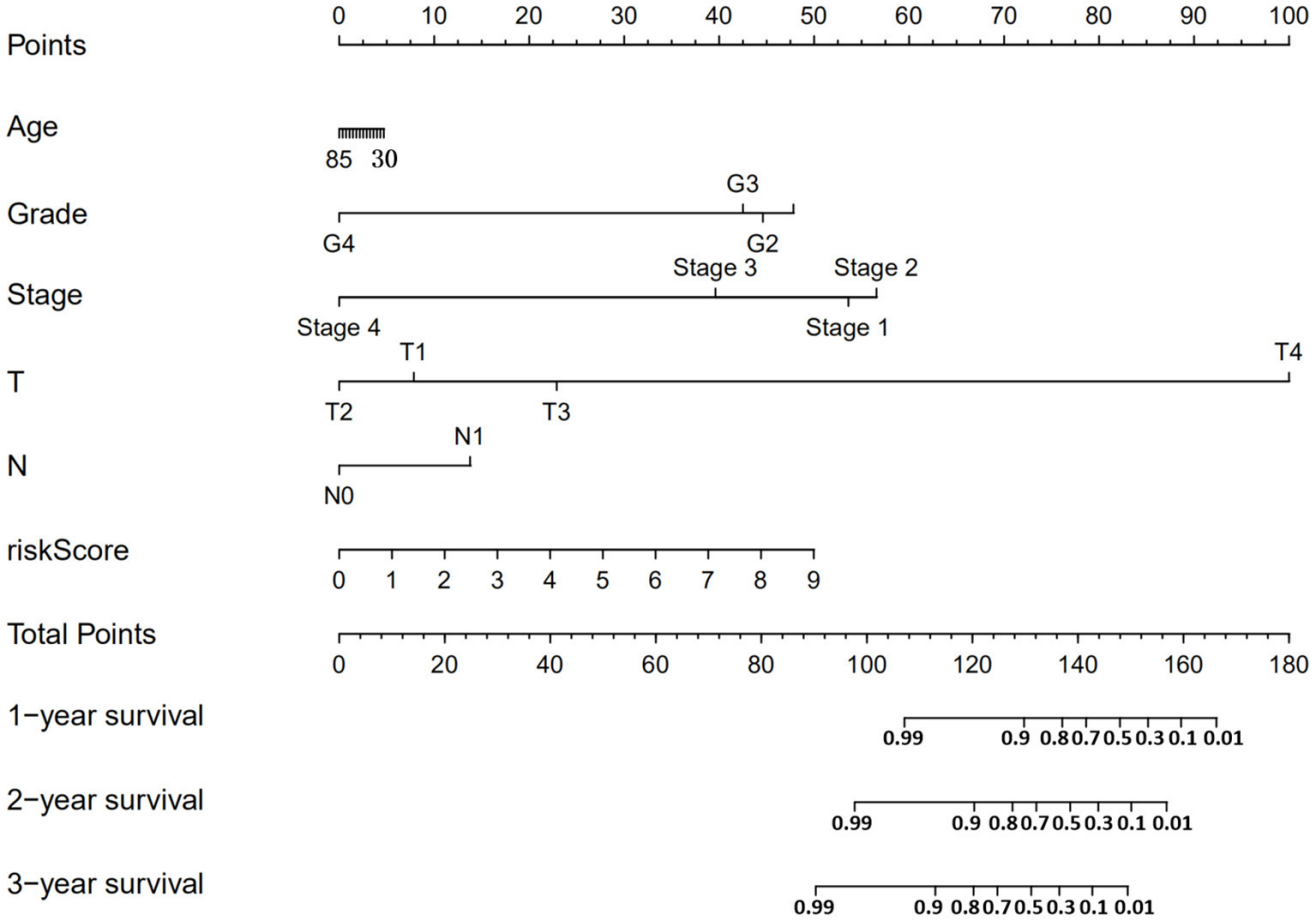
Nomogram can predict the 1,3, and 5 year overall survival of patients with CESC.

According to the risk score formula, the risk score of 306 CESC patients was calculated, and the median score was taken as the cut-off value. The patients were divided into high-risk group and the low-risk group, with 153 cases in each group (Figure 6A). The distribution of survival time shows that the number of deaths of CESC patients in the high-risk group is more than that in the low-risk group, and the patients with shorter overall survival (OS) in the high-risk group are more than those in the low-risk group (Figure 6C). Kaplan-Meier survival analysis was conducted for patients with high-risk and low-risk groups. The survival rate of low-risk group was significantly higher than that of high-risk group (Figure 6B). The results of ROC curve show that the AUC as the prediction efficiency of risk score for 1-year, 3-year and 5-year prognosis of patients are 0.786, 0.727, and 0.722 respectively, indicating that the model has a certain ability to predict the prognosis of CESC patients (Figure 6D). The nomogram provides a graphical representation of each factor. The prognostic risk of a single patient can be calculated from the points associated with each risk factor, which can be used to predict the 1, 3, and 5 year overall survival of CESC patients (Figure 7).

### Correlation Between Risk Score and Clinical Factors

**Figure 8:**
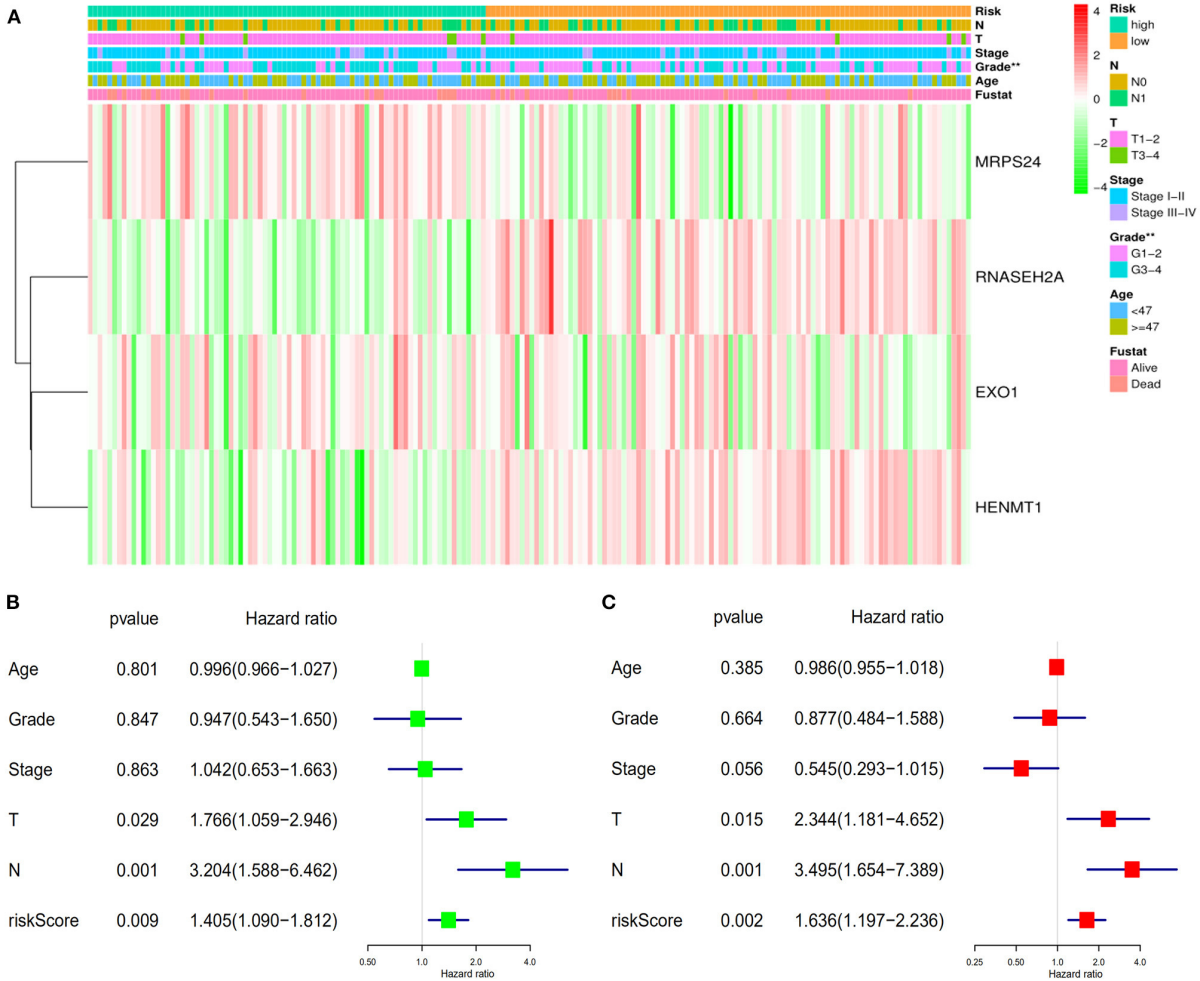
Verifies the accuracy of the prognostic scoring model. **(A)** Differences in the distribution of clinical factors between high and low risk groups. **(B)** Univariate prognostic analysis. **(C)** Multivariate independent prognostic analysis. **P < 0.01.

Data of TCGA-CESC data set were used to further study the correlation between patients' risk score and clinical factors in the model. Chi square test showed that there were differences in tumor grade distribution between high-risk group and low-risk group (Figure 8A). To assess whether the risk score was independent of other clinical variables, COX univariate and multivariate analyses were performed in 182 patients with complete clinical data. Univariate COX analysis showed that the T, N stages and riskScore of CESC patients were related to the prognosis (Figure 8B). Further multivariate COX regression analysis showed that riskScore, T stage and N stage were independent factors affecting OS in patients with CESC (Figure 8C).

### Validation of Prognostic Genes

**Figure 9:**
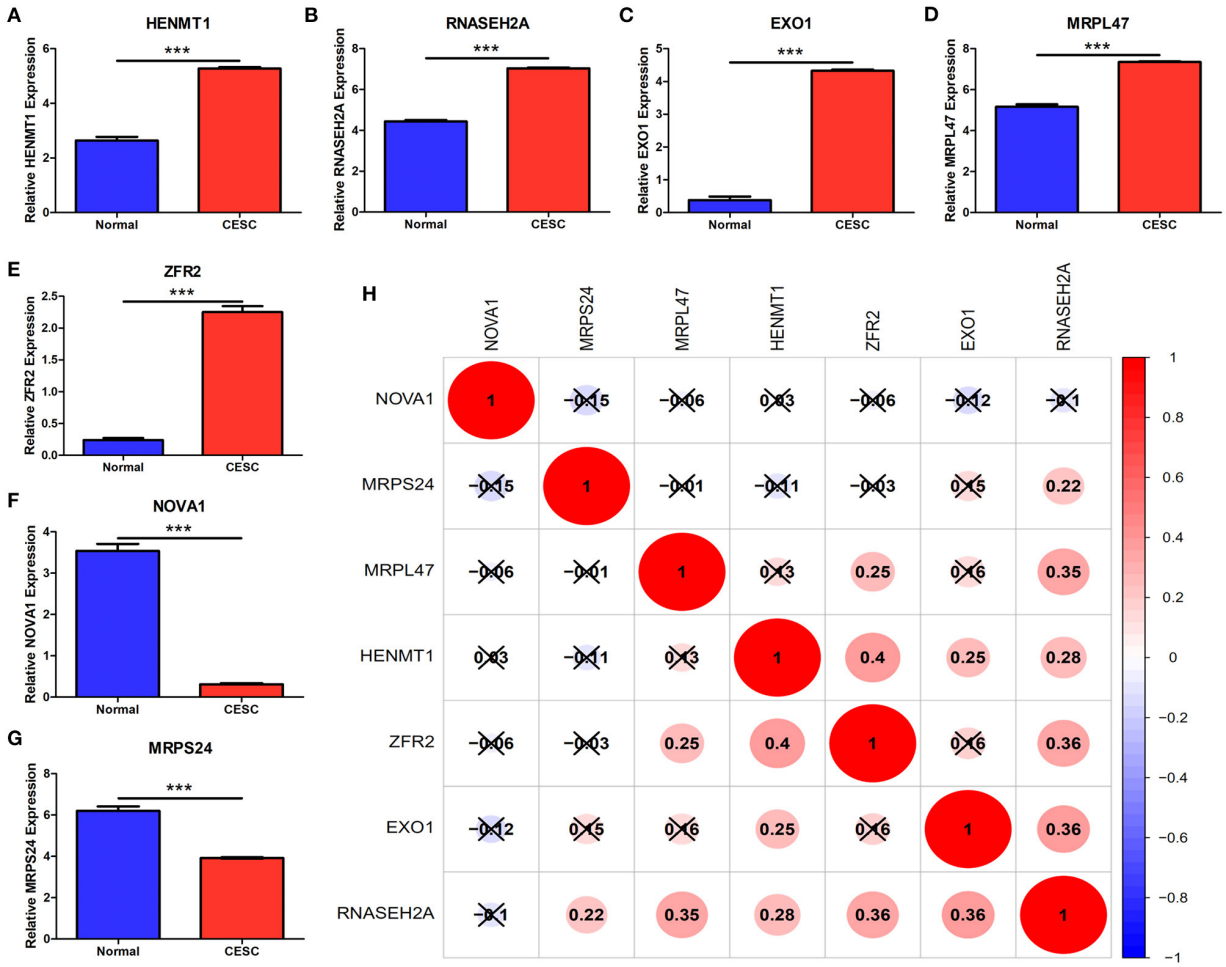
Differences in gene expression of seven RBPs between CESC and normal samples in the database. **(A)** HENMT1. **(B)** RNASEH2A. **(C)** EXO1. **(D)** MRPL47. **(E)** ZFR2. **(F)** NOVA1. **(G)** MRPS24. **(H)** Expression correlation analysis of seven RBPs. ***P < 0.001.

**Figure 10:**
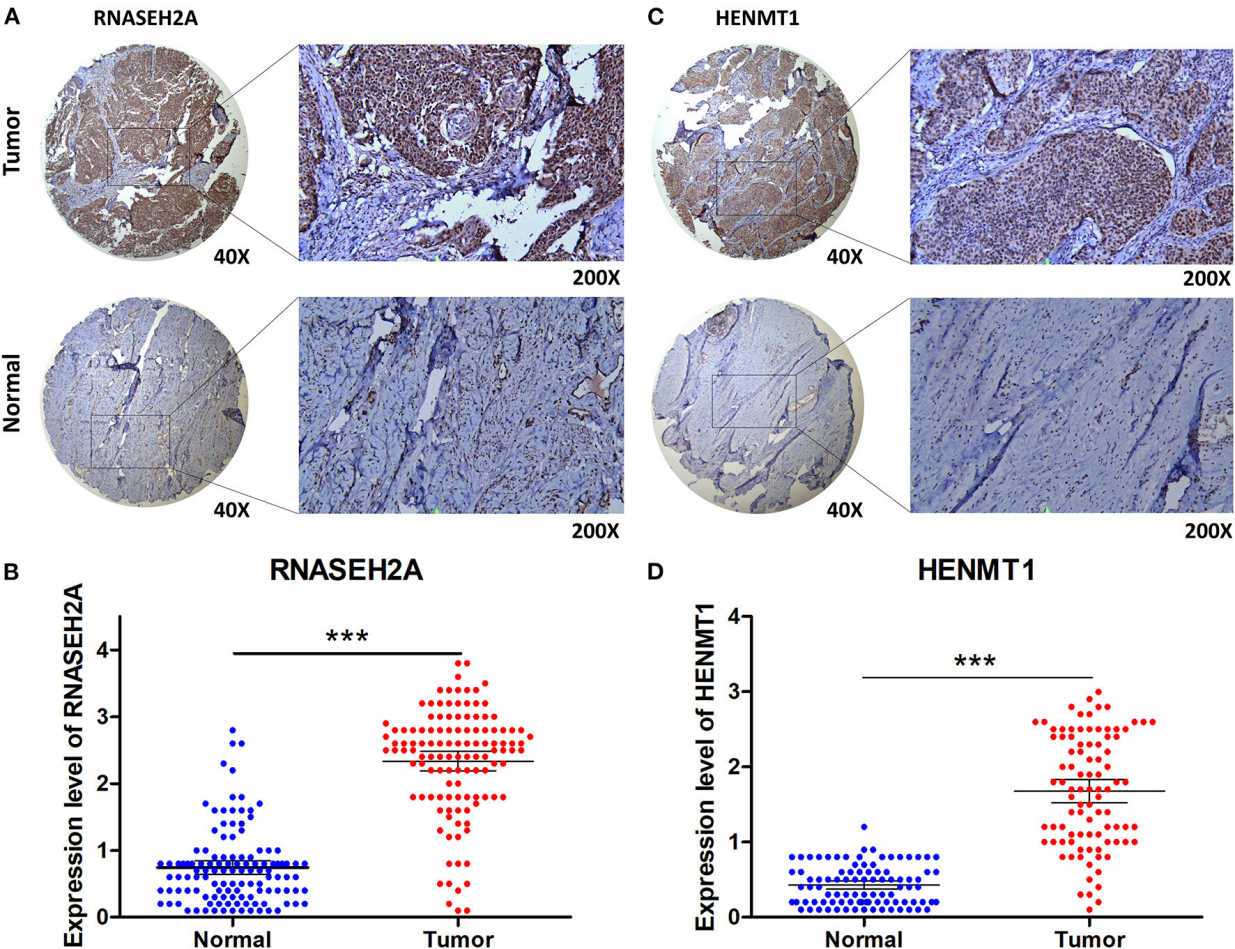
Immunohistochemical verification of RNASEH2A and HENMT1. **(A)** Expression of RNASEH2A protein in cancer and normal samples. **(B)** Differential expression analysis of RNASEH2A in cancer and adjacent tissues in tissue expression microarray. **(C)** Expression of HENMT1 protein in cancer and normal samples. **(D)** Differential expression analysis of HENMT1 in cancer and adjacent tissues in tissue expression microarray. *t-test* was used to compare the differential expression between cancer and adjacent samples. ***P < 0.001.

The expression data analysis of the database showed that there were significant differences in the expression of seven RBPs between CESC and normal tissues, including the up-regulated expression of five genes (HENMT1, RNASEH2A, EXO1, MRPL47 and ZFR2) and the down-regulated expression of two genes (MRPS24 and NOVA1) (Figures 9A–G). In addition, the expression of RNASEH2A was positively correlated with the expression of EXO1, ZFR2, HENMT1, MRPL47, and MRPS24. The expression of HENMT1 was positively correlated with the expression of EOX1 and ZFR2 (Figure 9H). Finally, protein expression levels of RNASEH2A and HENMT1 in cervical cancer and adjacent tissues were verified by immunohistochemistry. It can be seen that the expression of RNASEH2A and HENMT1 in tumor tissues is significantly higher than that in adjacent tissues (Figures 10A,C). In the RNASEH2A immunohistochemical expression micro array, after deleting the tissue points shed during the experiment, the expression of RNASEH2A in the remaining 120 pairs of cancer and adjacent tissues was statistically analyzed (Supplementary Figure 1). Statistical analysis showed that the expression of RNASEH2A in cervical cancer was higher than that in adjacent tissues (Figure 10B). In the HENMT1 immunohistochemical expression micro array, after deleting the tissue points shed during the experiment, the expression of HENMT1 in the remaining 93 pairs of cancer and adjacent tissues was statistically analyzed (Supplementary Figure 2). Statistical analysis showed that the expression of HENMT1 in cervical cancer was higher than that in adjacent tissues (Figure 10D).

### Correlation Between RNASEH2A and HENMT1 and Clinical Markers, and Evaluation of Diagnostic Effect

**Figure 11:**
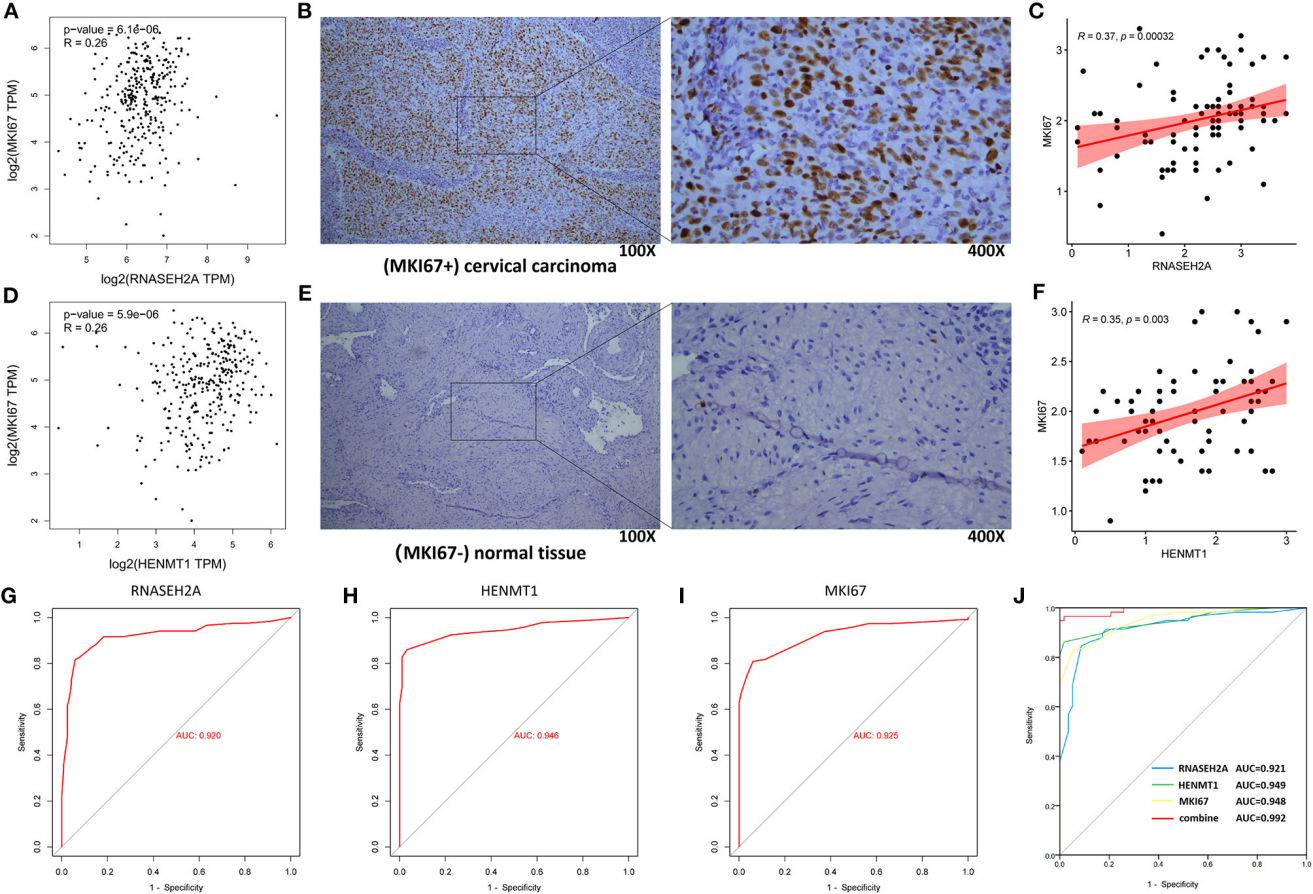
Correlation analysis between RNASEH2A and HENMT1 and clinical marker MKI67, and evaluation of molecular diagnostic effect. **(A,D)** The correlation between RNASEH2A and HENMT1 and clinical markers (MKI67) in CESC was analyzed by GEPIA database. R represents the correlation coefficient. **(B,E)** Expression of clinical marker (MKI67) in cervical cancer and normal tissues. The brown part represents the target protein. **(C)** The correlation between RNASEH2A and clinical marker (MKI67) was analyzed according to the immunohistochemical results. **(F)** The correlation between HENMT1 and clinical marker (MKI67) was analyzed according to the immunohistochemical results. **(G–I)** ROC curve was used to analyze the diagnostic effect of RNASEH2A, HENMT1 and MKI67 on cervical cancer. **(J)** The efficacy of RNASEH2A and HENMT1 combined with clinical marker (MKI67) in the diagnosis of cervical cancer.

GEPIA database analysis showed that RNASEH2A and HENMT1 were positively correlated with the expression of tumor marker (MKI67) (Figures 11A,D). Clinical immunohistochemical data showed that the expression of tumor marker (MKI67) in cancer tissues was stronger than that in normal tissues (Figures 11B,E). At the same time, RNASEH2A and HENMT1 were positively correlated with the expression of clinical markers (MKI67) in cancer tissues (Figures 11C,F). ROC curve analysis shows that the areas under RNASEH2A, HENMT1 and MKI67 curves are 0.92, 0.946, and 0.925, respectively (Figures 11G–I). However, RNASEH2A, HENMT1 combined with clinical markers (MKI67) calculated the largest area under the ROC curve, which was 0.992 (Figure 11J).

## Discussion

CESC is one of the most common malignant tumors in women worldwide, and has a high incidence rate (16). The risk of recurrence and metastasis of CESC is high. Early diagnosis and treatment are very important to improve the prognosis of CESC patients. Therefore, it is urgent to explore new potential biomarkers that can be used for early diagnosis, targeted therapy or prognosis evaluation to improve the prognosis of CESC patients. Micro array analysis is a high-throughput technology, which can detect the expression level of thousands of genes at the same time. Nowadays, abnormal gene expression is considered to be one of the factors in the occurrence and development of CESC, and more and more studies show that some deregulated genes in CESC may become candidate biomarkers for diagnosis and prognosis (17). RBPs are proteins that can bind to a variety of RNAs and can be stably expressed in cells. Their main role is RNA processing, such as mRNA splicing and translation regulation. In the past decades, it has been found that RBPs are closely related to the occurrence and development of many tumors (18). At present, narcotic drugs are prescription drugs for the treatment of cancer pain. Some narcotic drugs inhibit tumor growth, invasion and metastasis. Other narcotic drugs promote tumor growth, invasion and metastasis. Their mechanism may be related to regulating the immune ability of the body to the tumor. So choosing different anesthetic drugs in different preoperative periods may have different effects on tumor recurrence and invasion, and directly affect the prognosis of surgical patients. Therefore, this study analyzed the relationship between RBPs and narcotic drugs in CESC.

In this study, the gene expression profile of CESC was analyzed by bioinformatics to explore its molecular mechanism and identify important molecules that may be used as CESC biomarkers and therapeutic targets. In this study, we downloaded the gene expression profile data set of CESC from TCGA database, deeply analyzed it by bioinformatics method, and obtained the RBPs between CESC tissue and normal tissue. The results showed that 65 RBPs were identified between CESC and normal tissues. GO functional enrichment analysis showed that DEGs were mainly involved in ribosomal subunit, ribosome, large ribosomal subunit, structural constituent of ribosome, RNA splicing, RNA catabolic process, cytoplasmic translation, regulation of translation, double-stranded RNA binding and catalytic activity acting on RNA. In addition, enrichment results of KEGG pathway showed that the enriched pathways mainly involved Hepatitis C, Spliceosome and Ribosome. Post transcriptional regulation of RNA stability is an important step in the process of gene expression. RBPs can interact with RNA to form RNA protein complexes, so as to increase the stability of target mRNA, promote gene expression and play a key role in the progress of various diseases. The above information shows that our data mining results are consistent with the existing research results. In this study, local anesthetics (benzocaine, procaine, pentoxyverine and tetracaine) with regulatory function to 65 differentially expressed RBPs were analyzed. Among them, the regulation trend of benzocaine and tetracaine on genes is the same as that of RBPs. Although drugs have analgesic effect, they may lead to disease deterioration by promoting cell biological function. Procaine and Pentoxyverine may inhibit tumor progression on the basis of anesthesia and analgesia. Its effect on the tumor and its related mechanism needs to be further studied and discussed.

Survival analysis was used to screen the genes related to the prognosis of CESC patients in 65 differentially expressed RBPs. The results showed that seven RBPs (HENMT1, RNASEH2A, EXO1, MRPL47, ZFR2, NOVA1, and MRPS24) were related to the prognosis of patients. Among them, four genes (HENMT1, RNASEH2A, EXO1, and MRPS24) are independent predictors of the prognosis of CESC patients. Based on the above four genes, a CESC prognostic risk score model was constructed to further improve the reliability of the prediction results.

Hen methyltransferase 1 (HENMT1) is a methyltransferase. It is a kind of RNA composed of 24–30 nucleotides. It is produced by Dicer independent mechanism and mainly comes from transposons and other repeat elements (19, 20). The expression of HENMT1 in ovarian cancer is increased with the increase of tumor grade, which was related to the degree of malignancy (21). In this study, the expression of HENMT1 in cervical cancer patients is up-regulated, and its high expression indicates a better prognosis. As a low-risk gene, HENMT1 may be a marker for predicting the prognosis of cervical cancer patients.

Ribonuclease H2 subunit A (RNASEH2A) is an endonuclease that specifically degrades RNA. It can remove lag strand Okazaki fragment RNA primers by mediating the process of DNA replication. RNASEH2A participates in the occurrence of human glioma by promoting glioma cell proliferation and inhibiting apoptosis (22). Over expression of RNASEH2A is positively correlated with chemoresistance of breast cancer cells (23). RNASEH2A was highly expressed in lung cells, and its knockdown inhibited the proliferation of lung cells and induced apoptosis (24). Similarly, we found that RNASEH2A is highly expressed in cervical cancer and participates in RNA catabolic process, which is expected to become a molecular diagnostic marker and therapeutic target of cervical cancer.

Exonuclease 1 (EXO1) has both 5′-3′ exonuclease and 5′ structure specific endonuclease activities. It plays an important role in base mismatch repair, cross injury synthesis, nucleotide excision repair, DNA double strand break repair, meiotic recombination repair and telomere maintenance (25). It was found that the abnormal expression of EXO1 gene may affect the prognosis, survival and progress of patients with prostate cancer (26). The high expression of EXO1 in breast cancer and lung cancer may promote tumor development (27, 28). We found that EXO1 was highly expressed in cervical cancer and was verified by immunohistochemistry. EXO1 participates in catalytic activity in RNA and is expected to become a molecular diagnostic marker of CESC.

Mitochondrial ribosomal protein L47 (MRPL47) is a member of the MRPs family. Mitochondrial ribosome is composed of a small 28s subunit and a large 39s subunit. MRPL47 encodes a large subunit protein. MRPL47 gene mutation is a new high risk factor of vincristine induced peripheral neuropathy in children with acute lymphoblastic leukemia (29). MRPL47 was reported in square cell carcinoma of head and neck, which was related to the prognosis of patients (30). In this study, compared with normal samples, the expression of MRPL47 in CESC was up-regulated and involved in the structural components of ribosomes, and its increased expression in CESC showed a good prognosis.

Nova alternative splicing regulator 1 (NOVA1) is an RNA binding protein with a specific sequence. The protein has three KH type domains that can bind to RNA. In recent years, many studies have shown that NOVA1 is involved in regulating the occurrence and development of tumors. Zhang et al. (31) have shown that nova1 plays a carcinogenic role in primary liver cancer. Kim et al. (32) showed that the expression of NOVA1 was significantly down regulated in gastric cancer, and the low expression level of nova1 was closely related to the poor prognosis of patients with gastric cancer. Similarly, NOVA1 expression was down regulated in CESC, and patients with low expression had a poor prognosis. NOVA1 plays a role in RNA splicing and is expected to become a molecular diagnostic marker of CESC.

Mitochondrial ribosomal protein S24 (MRPS24) belongs to the universal ribosomal protein US3 family. At present, the role of MRPS24 in tumorigenesis and development has not been reported. In this study, the expression of MRPS24 was down regulated in CESC, which is the structural component of ribosome and a risk factor for poor prognosis.

Finally, in order to explore the correlation between the markers predicted in this study and clinical tumor markers, the correlation between the experimental results of HENMT1, RNASEH2A and clinical tumor markers was analyzed. It was found that HENMT1 and RNASEH2A were positively correlated with the expression of tumor marker (MKI67). At the same time, compared with the diagnostic efficiency of simple tumor markers, HENMT1 and RNASEH2A combined with clinical markers (MKI67) have higher diagnostic efficiency and reduce the misdiagnosis and missed diagnosis rate of patients to a certain extent.

In conclusion, we used bioinformatics methods to deeply mine the RBPs expression profile data set of CESC. On this basis, we mined narcotic drugs (benzocaine, procaine, pentoxyverine and tetracaine) that regulate RBPs. It was found that procaine and Pentoxyverine are expected to become potential drugs for the treatment of CESC. We screened seven hub genes (HENMT1, RNASEH2A, EXO1, MRPL47, ZFR2, NOVA1 and MRPS24). Among them, the prognostic risk model was constructed based on four independent predictors (HENMT1, RNASEH2A, EXO1 and MRPS24) of the prognosis of CESC patients. The model not only provides new insights into the heterogeneity of CESC, but also has independent predictive value for unconventional clinicopathological factors. It can provide patients with more accurate prognosis evaluation and individualized diagnosis and treatment.

## Data Availability Statement

The original contributions presented in the study are included in the article/Supplementary Material, further inquiries can be directed to the corresponding author/s.

## Ethics Statement

The studies involving human participants were reviewed and approved by the Affiliated Huaian No. 1 People's Hospital of Nanjing Medical University. The patients/participants provided their written informed consent to participate in this study.

## Author Contributions

YZ and JY participated in the study design, drafted the manuscript, and revised the manuscript. YZ and XM statistically analyzed the data. All authors read and approved the final manuscript.

## Funding

Huai'an Science and Technology support project, Grant No. HAB201927.

## Conflict of Interest

The authors declare that the research was conducted in the absence of any commercial or financial relationships that could be construed as a potential conflict of interest.

## Publisher's Note

All claims expressed in this article are solely those of the authors and do not necessarily represent those of their affiliated organizations, or those of the publisher, the editors and the reviewers. Any product that may be evaluated in this article, or claim that may be made by its manufacturer, is not guaranteed or endorsed by the publisher.

## Supplementary Material

Supplementary Table 1 Information list of 1,542 RBPs genes.

Supplementary Figure 1 The expression of RNASEH2A in the remaining 120 pairs of cancer and adjacent tissues.

Supplementary Figure 2 The expression of HENMT1 in the remaining 93 pairs of cancer and adjacent tissues.
