# Supplementary figures and images for: Exploring Potential Regulatory Anesthetic Drugs Based on RNA Binding Protein and Constructing CESC Prognosis Model: A Study Based on TCGA Database
Source: Front Surg. 2022 Apr 5;9:823566. doi: 10.3389/fsurg.2022.823566 (PMC9018109; doi:10.3389/fsurg.2022.823566)

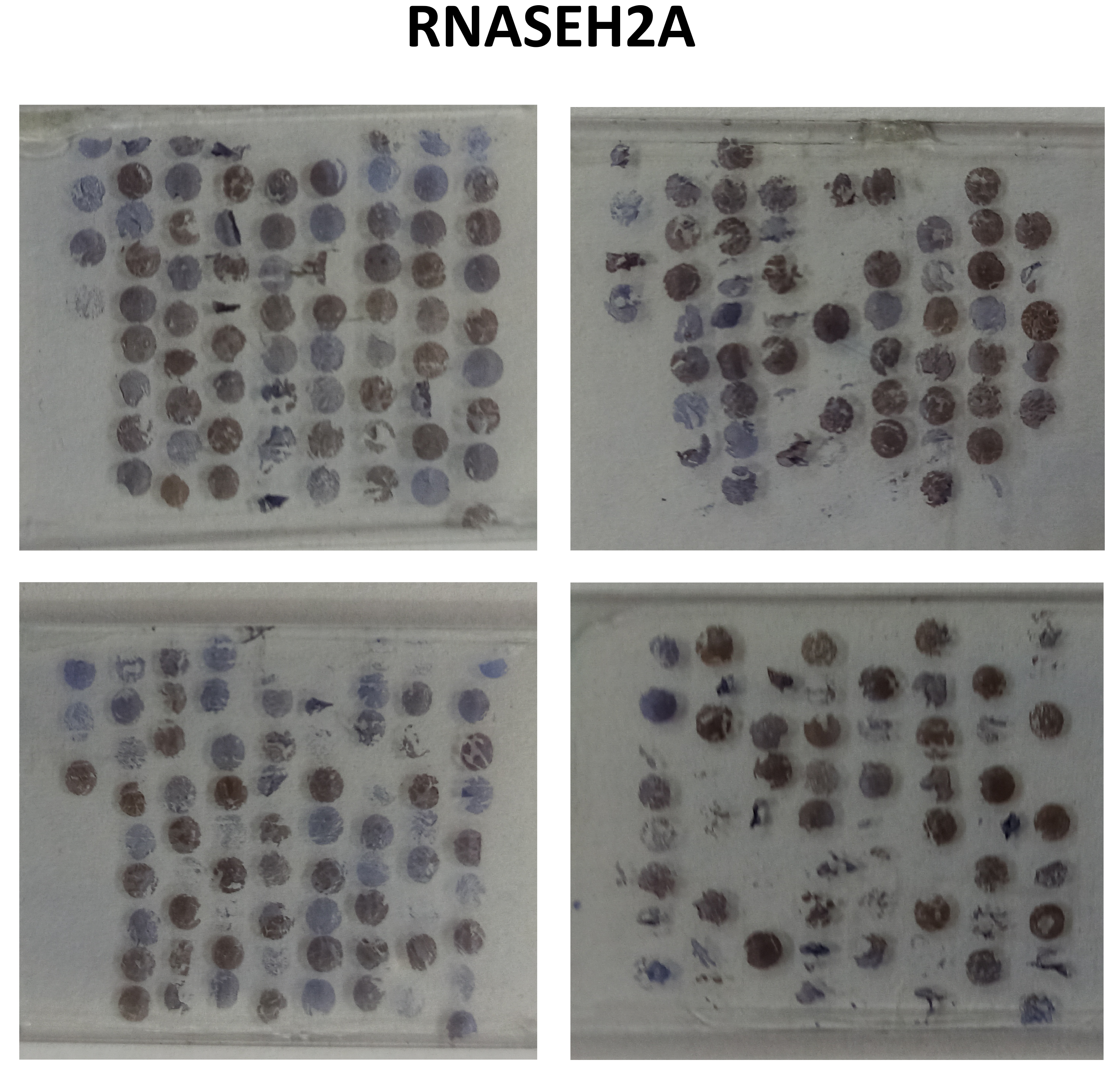

Supplement: Supplementary Figure 1 — The expression of RNASEH2A in the remaining 120 pairs of cancer and adjacent tissues. [file Image_1.TIF]

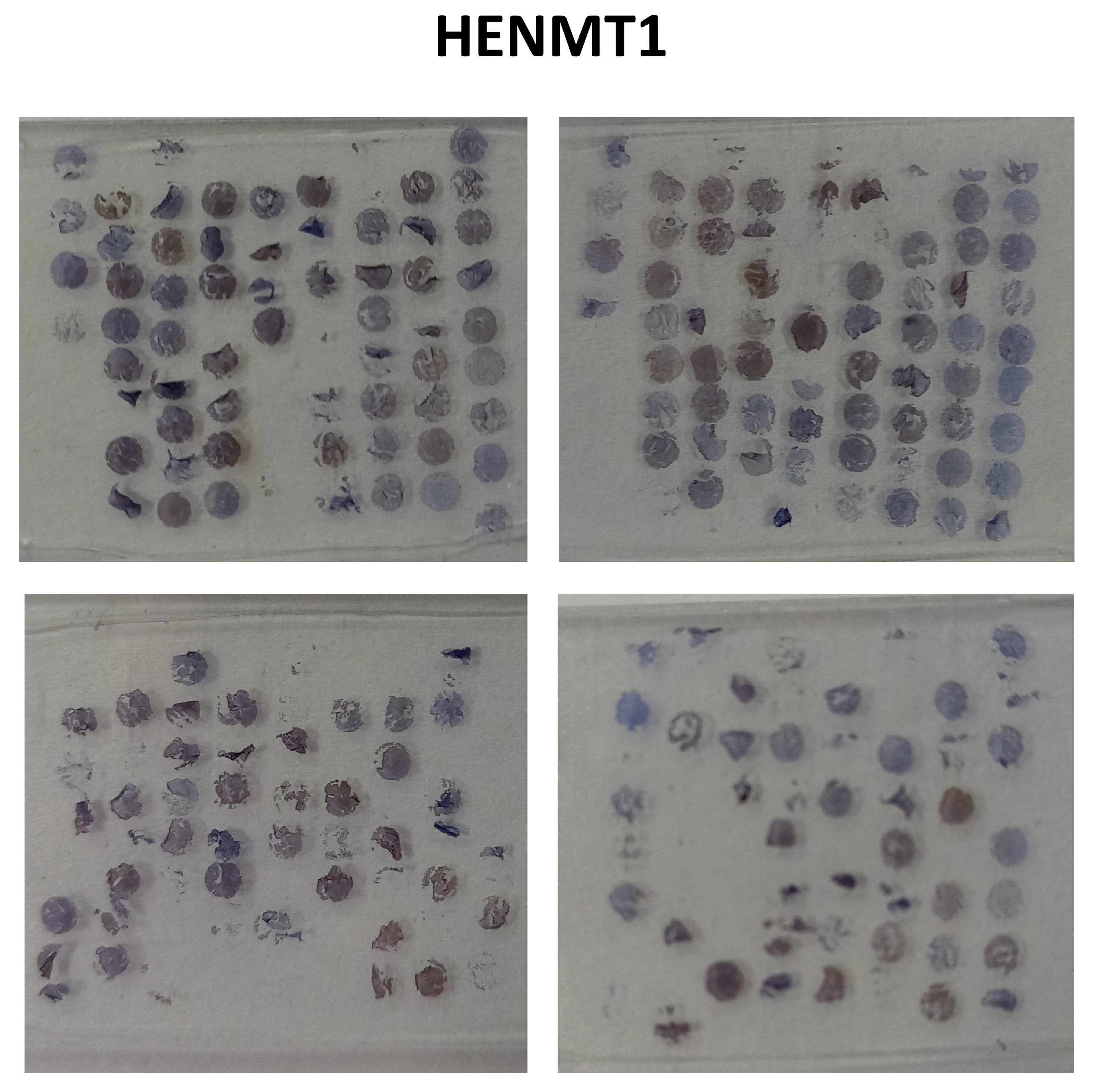

Supplement: Supplementary Figure 2 — The expression of HENMT1 in the remaining 93 pairs of cancer and adjacent tissues. [file Image_2.TIF]
